# Light-weights placed right: post-field constituents in heritage German

**DOI:** 10.3389/fpsyg.2023.1122129

**Published:** 2023-08-24

**Authors:** Wintai Tsehaye

**Affiliations:** Department of English Linguistics, University of Mannheim, Mannheim, Germany

**Keywords:** heritage German, right sentence periphery, post-field, light-weight constituents, German–English language contact, register

## Abstract

This study focuses on the linearization of constituents at the right sentence periphery in German, specifically on non-clausal light-weight constituents (LWCs) in the post-field. Spoken and written productions of German heritage speakers (HSs) with English as their majority language (ML) and of monolingually-raised speakers (MSs) of German are analyzed in different registers. The right sentence periphery is an area comprising a lot of variation and it is therefore intriguing to see how the two speaker groups deal with the options available if faced with the same communicative tasks. The overall goal is to answer the question whether the production of post-field LWCs in German HSs and MSs can provide us with evidence for ongoing internal language change and for the role of language contact with English. The analyses show a similar variational spectrum of LWC types and frequencies across speaker groups but a different distributional variation. The results show effects of register-levelling in the HS group, as they do not differentiate between the formal and informal setting unlike the MS group. Therefore, rather than transfer from the ML, the source of differing distributional variation of LWCs lies in the diverging adherence to register norms due to different exposure conditions across speaker groups.

## 1. Introduction

Heritage speakers (HSs) are a theoretically most relevant speaker group for linguistic research across subdomains of their grammars. Their often very heterogeneous acquisition context and outcome makes them an excellent learner type to investigate bilingualism, interface phenomena, as well as synchronic and diachronic effects of language contact. Heritage speaker’s linguistic competence and performance show considerable inter- and intraindividual variation and they often rate themselves better in spoken than in written productions (Montrul, 2016, p. 44ff.), especially where their heritage language (HL) is not supported within the educational system. Furthermore, specific linguistic areas are more prone to variation (e.g., morphology, discourse) than others (e.g., phonology, syntax). An explanation for variation across linguistic subdomains is found in the interface hypothesis (Sorace, 2011; Tsimpli, 2014), which states that “language structures involving an interface between syntax and other cognitive domains are less likely to be acquired completely than structures that do not involve this interface” (Sorace, 2011, p. 1).

**Figure 1:**
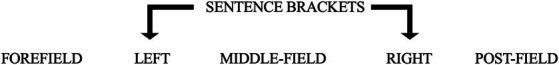
Illustration of the topological model.

Adopting a topological framework (see below), this paper focuses on the linearization of constituents at the right sentence periphery of German, specifically on post-field constituents in spoken and written productions of German HSs with English as their majority language (ML) and of monolingually-raised speakers (MSs) of German. I investigate the production of light-weight constituents (LWCs), i.e., non-clausal constituents which appear after the clause-final predicate, in the post-field (see Figure 1 and example 1). These particular clausal patterns diverge from the canonical pattern of German word order, and their status as more or less “marked” involves the interface of syntax and discourse-pragmatic factors.

In the topological model, constituents appear in different “fields” from which they can be moved either to the forefield, via topicalization, scrambled in the middle-field or extraposed into the post-field^1^ (Drach, 1963; Zifonun et al., 1997; Wöllstein, 2014; Zifonun, 2015). While the forefield and the left clausal edge have received considerable attention (Müller, 2003; Freywald et al., 2015; Wiese and Müller, 2018; Bunk, 2020; Rocker, 2022; Wiese et al., 2022, among others), less attention has been given to the post-field and the right clausal edge. Researchers who have however worked on the right sentence periphery have identified it as a very heterogeneous domain and called for a more differentiated analysis with conceptually separable subdivisions (see Vinckel-Roisin, 2015 for an overview).

In example (1), the LWC in the post-field is realized as the adverbial phrase (ADVP) *ganz schnell* (very quickly) which appears after the participle *gestoppt* (stopped).

(1) das erste Auto hat gestoppt (**ganz schnell**_**ADVP**_) (RUEG corpus informal spoken^2^)

“*The first car had stopped very quickly*.”

The post-field, broadly defined as the area following the right sentence bracket,^3^ is typically considered an area reserved for heavy constituents such as subordinate clauses extraposed from the middle-field in order to reduce cognitive load^4^ (Haider, 2010; Proske, 2015; Imo, 2016, p. 207). The realization of LWCs in the post-field as shown in example (1), while not ungrammatical, is often considered marked (e.g., Andersen, 2008; Vinckel-Roisin, 2012; Frey, 2015 among others). However, when we take into account different registers in speaking and writing, the situation is not as straightforward. Depending on the formality and mode of a production, we find a considerable range of constituents, like those in example (1), in the post-field not only of HSs but also of MSs of German. Therefore, the role and the effects of register variation need to be included in the analysis of LWCs in the post-field.

Previous research has shown that prepositional phrases (PPs) are particularly frequent in the post-field (Haider, 2010, p. 191; Zifonun, 2015; Imo, 2016). In German, PPs can occur before the verb, in the middle-field (example 2a), or after the verb, in the post-field^5^ (example 2b).

(2a) weil das Auto (**wegen dem Hund**_**PP**_) stoppen musste.

(2b) weil das Auto stoppen musste (**wegen dem Hund**_**PP**_).

“*Because the car had to brake on account of the dog*.”

In English, comparable PPs must follow the verb but cannot appear between the subject and the verb. Therefore, within the analysis of LWCs undertaken here, special emphasis is placed on the extraposition of PPs across speaker groups as it can provide us with information on the influence of language contact.

Even though English and German are both Germanic languages, they exhibit considerable typological differences in terms of word order. These differences make English and German an intriguing language pair to investigate the influence of language contact, language dominance and transfer potential. German is among one of the better-researched languages in the field of HL research. There is a long-standing history of investigations on Germanic varieties in English dominant environments, such as Australian German, Texas German, Pennsylvania German, and Moundridge Schweitzer German and existing research on these varieties, indeed, finds trends of increased frequencies of LWCs in the post-field attributable to language contact with English (e.g., Clyne, 2003; Westphal Fitch, 2011). However, there is so far little work on the type of HSs discussed here, namely second-generation immigrants born in the U.S. or early-childhood arrivers who are not part of a bigger German speaking Language Island community.

Overall, the phenomena investigated here have until recently been neglected in German linguistics, under-researched for different acquisition types, and, to the best of my knowledge, not pursued in research on German as a HL in second-generation immigrants under intense language contact with English as a ML. Section 2 provides the theoretical background and anchors the present analysis in previous studies. Section 3 introduces the participants, the corpus, and the applied methodology. Section 4 illustrates the results, followed by a discussion in Section 5. Section 6 summarizes the results and addresses limitations of the current analysis as well as perspectives for follow-up research.

## 2. Theoretical background

### 2.1. Heritage speakers

One finds a plethora of HS definitions in the literature, depending on the theoretical research focus. According to the definition adopted here, HSs are bilinguals who grow up acquiring their HL within the family but are raised in an environment where another language has majority status (Rothman, 2007; Montrul, 2016; Polinsky, 2018). They can be considered either simultaneous bilinguals, exposed to two languages (the HL and the ML) from birth, or early sequential bilinguals who first acquire the HL and are then exposed to the ML of their country of residence. Intensive exposure to an early second language often results in a dominance shift from the HL to the ML (Pascual Y Cabo and Rothman, 2012; Kupisch and Rothman, 2018; Ortega, 2020 among others). Consequently, HSs usually use their ML in a wider range of communicative situations than their HL. In some cases, they may only be addressed in their HL by one other family member, in other cases, there may be an actual HL speaker community outside the family.^6^

Past research on HSs reveals a deficit-oriented view on their linguistic competence and performance, which resulted in labels such as semi-speakers or incomplete acquirers. However, this view has shifted due to a surge of interest in divergent attainment or differential acquisition (cf. Kupisch and Rothman, 2018) and led to extensive discussions of a suitable baseline, i.e., the actual input that HSs receive in the HL and not the variety spoken by MSs they are not exposed to (Polinsky, 2018, p. 3ff.; Rothman et al., 2022). Accordingly, recent studies argue that HSs are native speakers of their HL (Rothman and Treffers-Daller, 2014; Montrul, 2016; Kupisch and Rothman, 2018; Tsehaye et al., 2021; Wiese et al., 2022). In the current study, the data collected from German MSs is not used as a baseline, but as comparative data enabling us to identify contact-independent internal dynamics as well.

### 2.2. Syntactic linearization in German

The topological model, first conceptualized by Drach (1963), uses the metaphors of *sentence brackets* and *topological fields* to describe and investigate German sentences. It should be emphasized that using the topological model results in a purely linear analysis and not in hierarchical, binary-branching structures.^7^ Table 1 illustrates the placement of constituents across topological fields with unmarked post-field constituents.

**Table 1:** Example sentences with unmarked post-field constituents.

**3**	**Forefield**	**Left sentence bracket**	**Middle-field**	**Right sentence bracket**	**Post-field**
a	Ich	habe	heute einen ziemlich heftigen Unfall	erlebt.^1^	
	*‘I have experienced a rather severe accident today.*				
b	Ich	wollte	gerne über einen Unfall	berichten	den ich gesehen habe.
	*‘I would like to report about an accident which I have seen.’*				
c	den		ich	gesehen habe.	
	*‘which I have seen’*				
d	Ich	wollte	gerne über einen Unfall, den ich heute gesehen habe,	berichten.	
	*‘I would like to report about an accident which I have seen today.’*				
e	Dann	fingen	die beiden Autofahrer	an,	den Unfall zu begutachten.
	*‘Then both drivers started to assess the accident.’*				

1 Most of the examples throughout this article have been taken from the RUEG corpus and were indicated as such (https://korpling.german.hu-berlin.de/annis3/#c=rueg). Some of the examples have been adapted to illustrate the variational spectrum of German sentences. They do, however mirror the syntactic patterns identified in the corpus.

In main and declarative clauses (examples 3a/b/d/e) the finite verb occurs in the left sentence bracket (LSB) while the rest of the verbal complex occurs in the right sentence bracket (RSB). In subordinate clauses (example 3c), complementizers^8^ occupy the LSB while the finite predicate occurs in the RSB. The area in front of the LSB is called the forefield. It holds constituents that are pre-posed or topicalized from the middle-field, which is the field encompassed by the sentence brackets. The area after the RSB is labeled the post-field. The post-field can hold constituents that have been extraposed from the middle-field, including clausal adjuncts such as relative or complement clauses (see examples 3b/e).^9^ While Table 1 showed the canonical, unmarked linearization of constituents in German sentences, Table 2 illustrates a different set of cases, thereby shifting the attention to the spectrum of constituents found in the post-field.

**Table 2:** Example sentences with marked post-field constituents.

**4**	**Forefield**	**LSB**	**Middle-field**	**RSB**	**Post-field**
a	Ich	habe	heute einen Unfall	beobachtet	auf einem Parkplatz.
	*‘I have observed an accident in a parking lot today.’*				
b	Ich	habe	einen ziemlich heftigen Unfall	beobachted	heute.
	*‘I have observed a rather severe accident today.’*				
c	Ich	habe	heute einen Unfall auf einem Parkplatz	beobachtet	einen ziemlich heftigen.
	*‘I have observed a rather severe accident in a parking lot today.’*				

Although the clauses in Table 2 show canonical verb placement, we also see deviations from what are assumed to be orthodox—or stylistically “desirable”—constituent candidates in the respective fields. Example (4a) illustrates the extraposition of the PP *auf einem Parkplatz* (in a parking lot). Example (4b) exhibits the placement of the adverbial *heute* (today) in the post-field while example (4c) shows the extraposition of the DP *einen ziemlich heftigen* (a rather severe one).

All post-field constituents in Table 2 can be categorized as LWCs which, as in the case of (4a/b) could have easily “stayed” in the middle-field. Example (4c) functions as the specification of the DP antecedent *einen Unfall* (an accident) in the middle-field and, thus, could not have been realized in the middle-field. However, the DP could have been modified as *einen ziemlich heftigen Unfall* (a rather severe accident) within the middle-field, i.e., there is no syntactic demand to extrapose this information. Such occurrences show the existence of a variational spectrum that holds especially for spoken productions of German (cf. Zifonun et al., 1997; Imo, 2015; Zifonun, 2015). A greater variational spectrum in spoken or conceptually spoken^10^ productions compared to written or conceptually written productions has been shown for other syntactic phenomena as well, suggesting that some linearization patterns might occur exclusively or more frequently in the spoken mode (Andersen, 2008, p. 2). However, variation is also found in written productions. Previous studies have attested considerable variation in the frequency of post-field productions in the written mode, with the least occurrences in scientific texts and most occurrences in informal productions (Roelcke, 1997, p. 158). This strengthens the fact that register differentiations need to be taken into account in investigations of post-field variation.

The availability of large synchronic and diachronic corpora of spoken and written German shows that even across MSs of German, the right sentence periphery is an area of considerable variation, with fluctuating degrees of markedness across registers. It is therefore intriguing to ask how both speaker groups, HSs and MSs, when faced with the same communicative challenge, deal with post-field options, given the fact that HSs of German have less contact with different registers than MSs and experience extensive language contact.

The existence of a post-field and its availability for various constituents in it is ultimately dependent on the formation of the sentence brackets. Only after the distinction of finite and non-finite verbs, and the asymmetric placement of finite and non-finite verbs in main and subordinate clauses is mastered, are we able to assess whether and with which constituents the post-field is filled. Head directionality within the verb phrase (VP), and hence, the RSB, are acquired early in L1, quickly followed by the discovery of the LSB and its canonical occupant, finite verbs (Tracy, 2011; Schulz and Tracy, 2018). The head parameters relevant for German main and subordinate clauses can be considered fixed around age three (Fritzenschaft et al., 1990; Rothweiler, 2006; Tracy, 2011; Müller et al., 2018). Once the post-field “exists”, learners still need to figure out which constituents can access it. A study which looked at the emergence of the topological fields and the occurrence of constituents in the right sentence periphery in children around age two found instances of complements, i.e., direct objects in form of DPs, in the post-field, which is highly non-canonical in contemporary German. With time, children’s productions converged on those of adults and became canonical (Elsner, 2015). The results of this study illustrate that even in monolingual L1 acquisition without contact with another language, one finds (non-) canonical variation in the linearization at the right sentence periphery.

After head directionality and finiteness are acquired, the placement of constituents in the post-field is furthermore influenced by register norms and discourse-pragmatic requirements of the communicative situation which will be outlined in the following. According to Biber and Conrad (2001, p. 175), a register is a variety which can be defined by specific communicative and contextual parameters, such as interlocutors involved, purpose, as well as mode and formality of the interaction. Previous research (Polinsky, 2018, pp. 323–324; Aalberse et al., 2019, p.148 to name but a few) has shown that HSs, who often do not learn to read and write in the HL, cannot be expected to have available the register spectrum, genres, or styles accessible to age-matched ML speakers of the same language in the country of origin. Dominance shift, the unavailability of a HL community, the greater social prestige of their ML, as well as the absence of formal education in the HL contribute to diverging levels of adherence to register norms between HSs and MSs as well as between the HL and the ML in individual speakers.

Discourse-pragmatic reasons for placing constituents in the post-field are manifold, and arguments for differentiating various subfields and ways for filling them (movement, free adjunction) are controversial, as shown in previous research (Zifonun et al., 1997; Frey, 2015; Vinckel-Roisin, 2015; Zifonun, 2015; Imo, 2016, among others). It has been argued that (a) the post-field cannot be a single undifferentiated field^11^ and (b) not all constituents that appear in this area seem to be extraposed from the middle-field but could also be more or less freely adjoined and base-generated (Vinckel-Roisin, 2012; Frey, 2015). Zifonun et al. (1997) propose subdividing the right sentence periphery into two fields: the post-field and the right outer field. The post-field contains syntactically integrated as well as non-integrated constituents such as subordinate clauses. The right outer field can be distinguished from the post-field insofar as its constituents are not syntactically integrated units of the preceding clause (Vinckel-Roisin, 2012). The right outer field can be occupied, regardless of whether or not the post-field is filled, and constituents in this position are typically prosodically or orthographically highlighted. The right outer field is usually reserved for constituents with discourse-pragmatic functions such as comments, verification of the audience’s attention or requests for reactions (cf. Imo, 2016, p. 223 ff.) Example (5) illustrates this distinction with the relative clause *der ziemlich heftig war* (which was rather severe) in the post-field and the discourse marker *nicht wahr* (isn’t that right) in the right outer field.

(5) Wir haben heute einen Unfall auf einem Parkplatz gesehen, der ziemlich heftig war, **nicht wahr**?

“*We saw an accident in a parking lot today, which was rather severe, isn’t that right*?”

Depending on their placement within the overall area of the post-field (narrow vs. extended post-field), their clausal status, and the degree of phonetic integration,^12^ functions addressed in the literature on MSs of German are the addition of detail to previously mentioned content, repairs, and evaluative afterthoughts in the service of discourse coherence.^13^

### 2.3. The influence of language contact

As already mentioned, the HSs in this study have English as their ML. For the phenomena under discussion in this paper, the most crucial difference between German and English consists in verb placement, with German being head-last within the VP, while English is head-first. German further exhibits an asymmetry in finite verb placement, with V2 structures in main clauses and VE structures in subordinate clauses, whereas English has an SVO structure across clauses apart from subject-auxiliary-inversion and highly restricted subject-main-verb-inversion with intransitive verbs (see Table 3).

**Table 3:** German and English word order.

	Contrasts	German	English
I	VP (across clauses)	[O....V_(-fin)_]	[V_(-fin)_ O …]
II	main clauses	(X) V2_(+fin)_ ….V_(-fin)_	(X) SV_(+fin)_O + residual V2
III	subordinate clauses	COMP...... V_(+fin)_	COMP SV_(+fin)_O

One relevant question to ask, then, is the following: Given intensive language contact between German and English, to what extent do HSs observe these contrasts? Do we see an increase in extrapositions which could be due to cross-linguistic influence from English? Such trends have been observed in previous studies on speakers of German Language Islands. Westphal Fitch (2011) found increased numbers of extrapositions in spoken productions in speakers of Palatinate and Pennsylvania German in comparison to speakers of Standard German due to language contact with English.

Despite the variational spectrum documented especially in spoken German, a crucial restriction, as already mentioned, is that contemporary German, does not allow the placement of direct objects in the post-field^14^ (Zifonun, 2015, p. 30), as in example (6).

(6) *Wir haben gesehen **einen Hund**.

“*We have seen a dog*.”

The translation of example (6) demonstrates that English calls exactly for this linearization, with the verbal head immediately adjacent to its complement. Previous studies on heritage German in Australia also attested increased extrapositions of LWCs, including the extraposition of direct objects, which Clyne (2003), attributes to intense contact with English, see example (7).

(7) Mummy hat gesagt **die Wörter für mich**.

“*Mummy told me what to say*”(Clyne, 2003, p. 137).

Productions like the one in example (7) legitimize the question whether language contact with English enhances the non-canonical placement of direct objects in the post-field of HSs of German.

The typological differences between English and German also become apparent when looking at the linearization of PPs. In English for instance, PPs usually appear after the verb due to the strict VO serialization across clauses.^15^ In German, due to the sentence brackets, the PP can occur in the middle-field (i.e., before the finite verb) or in the post-field (i.e., after the finite verb). Therefore, HSs have an additional option for PP placement in German in comparison to English. Choosing to extrapose the PP into the post-field results in clauses which are, in their surface syntactic realization, more parallel to the unmarked English linearization contrary to producing the PP in the middle-field, which is not possible in English. Research on German Language Islands in the USA has shown that if parallelism between structures exists, these structures may appear more frequently than non-parallel ones (Westphal Fitch, 2011, p. 374; Hopp and Putnam, 2015 and the references therein).^16^

Examples (8a/b) were produced by the same participant, once in the HL, German and once in the ML, English and illustrate this surface parallelism with the PP following the verb in both cases.

(8a) der Hund an der anderen Seite von der Straße ist vorgerannt (**zum Ball**_**PP**_) RUEG corpus formal written.

“*The dog on the other side of the street ran towards the ball*.”

(8b) and the dog leaped forward (**to the ball**_**PP**_) RUEG corpus formal written.

In the light of this typological difference between German and English, the question arises whether language contact with English facilitates the production of PPs in the post-field of German HSs, resulting in an overlapping surface structure across their languages—a question that explores the interplay of surface parallelism on the one hand and transfer or avoidance on the other hand.

An additional point—and analytical problem—paramount to the question of cross-linguistic influence and transfer phenomena due to surface parallelism is the fact that whenever we have a clause with an empty RSB (9a) or a clause with an empty RSB and a filled post-field (9b), the surface structure between German and English clauses becomes identical (see Table 4).

**Table 4:** Example clauses with empty RSB illustrating surface parallelism.

9	Forefield	LSB	Middle-field	RSB	Post-field
a	Ich	sah	einen Autounfall.	-	
	*‘I saw a car accident.’*				
b	Ich	sah	einen Autounfall	-	gestern.
	*‘I saw a car accident yesterday.*				

In the face of these partial overlaps and cross-linguistic parallels in surface structure, the question of whether contact with English boosts LWCs (including direct objects) in the post-field in HSs in comparison with MSs becomes particularly relevant.

### 2.4. The present study

The data presented in this article was not specifically elicited to investigate post-field productions. Nevertheless, it is highly suitable to investigate the variational spectrum at the right sentence periphery in different registers and the role of language contact: It contains the productions of MSs and HSs of German who were faced with the same communicative tasks, therefore allowing for adequate comparisons. The following research questions and hypotheses could therefore be formulated:

*RQ1*: Which types of LWCs can be found in the post-field of HSs and MSs of German, and with which frequency?

*H1*: Due to typological differences in the syntactic realization of constituents in German and English, HSs will show more various LWCs and increased frequencies of LWCs in their post-field productions.

*RQ2*: Does register influence the type and frequency of constituents in the post-field of HSs and MSs of German?

*H2*: Register will have an influence on the frequency of LWCs in the post-field across speaker groups with more constituents produced in the informal setting and the spoken mode.

*RQ3*: Do HSs of German produce more PPs in the post-field than MSs of German?

*H3*: HSs of German will have higher frequencies of PPs in their post-field than MSs of German due to extensive contact with English.

## 3. Method

### 3.1. Participants

The present study included 61 adolescent participants aged 13 to 19 years (mean age = 16.1, SD =1.35, 32 females). The overall number of participants can be subdivided into 29 HSs of German with ML English (mean age = 15.6, SD = 1.57, 12 females),^17^ and 32 MSs of German (mean age = 16.6, SD = 0.91, 20 females). All HSs grew up in the USA in a majority English environment, speaking German with at least one native German-speaking parent in the household.^18^ The participants in the MS group were defined as individuals whose L1, German, was the only language spoken at home, but who might have acquired further languages through foreign language instruction. The German and English productions of the HSs were elicited in the U.S., the productions of the German MSs in Germany. The data was retrieved from the openly accessible RUEG 0.4.0 corpus (Wiese et al., 2021).

### 3.2. Materials and procedure

The controlled and standardized data elicitation followed the language situations methodology (Wiese, 2020). Participants watched a short non-verbal video of a rear-ending car accident and recounted what they saw, imagining themselves witnesses to the accident in four different narrations, which we operationalized as productions in different registers. Data collection took place in two differently arranged rooms: a formal and an informal one with different elicitors in each room. The elicitation of the formal productions took place in an office-like room, whereas the informal productions were elicited in a casual setting with snacks and beverages offered and following a 10–15 minute-long informal, task-unrelated conversation in the target language in order to create a more relaxed atmosphere. During one session, all participants watched the video three times in total (twice in the first setting, once in the second setting) and were asked to recount it in two different modes: spoken and written.

In the formal recounting, the participants were asked to send a voice message to a police hotline (spoken) and a witness report to the police (written). In the informal setting, they had to send a voice message (spoken) and a text message (written) to a friend via an instant messenger. The order of settings (formal/informal) and modes (spoken/written) was balanced across participants. The MSs completed all tasks in one session. The HSs completed the tasks in two sessions – one for each language – with an interval of three to five days in between to minimize priming effects and the order of languages counterbalanced across participants. Upon completion of all tasks, participants filled out an online questionnaire^19^ about their language background as well as a self-assessment of their abilities in each language on a five-point Likert scale. Self-assessment showed that, in line with previous research, HSs rated their speaking skills higher than their writing skills in their heritage German (speaking mean = 3.71, SD = 0.79; writing mean = 3.03, SD = 1.29). German MSs rated their speaking skills at ceiling and their writing skills almost at ceiling (speaking mean = 4.96, SD = 0.17; writing mean = 4.6, SD = 0.64).

### 3.3. Data analysis

The spoken and written productions of both speaker groups (HSs and MSs) were annotated according to the topological model based on the KiDKo annotation guidelines (Bunk et al., 2020). All post-field constituents were exported from the RUEG corpus and additionally annotated for their constituent type. Table 5 shows examples for each constituent type produced in the post-field. A total of 708 post-field constituents were annotated.

**Table 5:** List of constituents in the right sentence periphery with examples.

Constituent type	Example
SC: subordinate clause (finite)	hat den mann nicht gesehen ***[weil ein auto in sein sichtfeld war***_***SC***_*]**^1^* *‘didn’t see the man because a car was in his field of view’*
INF: subordinate clause (non-finite)	und ein hund hat versucht ***[ihn zu fangen***_***INF***_***]*** *‘and a dog tried to catch it’*
PP: prepositional phrase	die haben die Straße runtergelaufen ***[mit einem Ball***_***PP***_***]*** *‘they walked down the street with a ball’*
ADVP: adverbial phrase	das auto vorne hat angehalten ***[plötzlich***_***ADVP***_***]*** *‘the car in front had stopped suddenly’*
DP: determiner phrase	die haben irgendwelche Sachen fallen gelassen ***[Lebensmittel***_***DP***_***]*** *‘they have dropped some things, groceries’*
ADJP: adjectival phrase	und die Frau war sehr schockiert **[also bisschen perplex**_**ADJP**_***]*** *‘and the woman was very shocked so a bit perplexed’*
DM: discourse marker	und die autofahrer sind dann auch gleich ausgestiegen ***[und so***_***DM***_***]*** *‘and the drivers immediately exited and so on’*
NONC: non-canonical direct object	die Mann geht zu helfen ***[die Mädchen [die essen aufzuholen]***_***NONC***_***]*** *‘the man goes to help the girl pick up the food’*

1 All productions in this table have been kept in their original orthography, if written, and in their original structure, if spoken, while canonical morphosyntax and choice of auxiliary have been ignored.

The corpus includes a total of eight different constituent types: finite subordinate clause (SC), non-finite subordinate clause (INF), prepositional phrase (PP), adverbial phrase (ADVP), determiner phrase (DP), adjectival phrase (ADJP), discourse marker (DM), and DP realized as non-canonical direct object (NONC) of which we found a total of two in the corpus, both produced by the same speaker.

As has already been established, the occurrence of (non-)finite subordinate clauses in the right sentence periphery is canonical and unmarked as it serves to avoid “overloading” the middle-field. Therefore, the focus of the current analysis lies on constituents that are not subordinations, i.e., LWCs. Due to scarceness of data points (a total of 140 LWCs) and, therefore, small numbers in certain categories, the eight constituent types were collapsed into subordinations and LWCs. This resulted in a dependent variable “constituent type” with two levels (1 for LWCs and 0 for SCs^20^). Generalized binomial linear mixed effects models in R (R Core Team, 2021) and the lme 4 package (Bates et al., 2015) were used to analyze the distribution and frequency of LWCs in the right sentence periphery. I specified the fixed effects by including the following dependent variables and their potential interactions: speaker group (HS/MS), setting (formal/informal), and mode (spoken/written) and I used treatment contrast and maximally specified the random effect of participants. To avoid overfitting, I performed backward ANOVAs to deduce the most suitable model. For each model, the *z*- and *p*-values are reported.

In order to answer the third research question, I additionally performed an analysis on the distribution of PPs across narratives and speaker groups. The dependent variable for this analysis was “PP” with two levels (1 for PP and 0 for no PP). Again, I maximally specified the fixed and random effects, used generalized binomial linear mixed effects models, and performed backward ANOVAs for model fitting.

The language situations method and the included task of recounting an accident, especially where a police report is called for, creates a bias in favor of a specific functional kind of extrapositions, namely providing expansions or specifications. Therefore, the post-field constituents can be categorized as:

- constituents that can be placed in the middle-field or the post-field resulting in different degrees of markedness: less marked for extraposed heavy constituents such as subordinations with the function of decreasing cognitive load, and more marked for LWCs functioning as afterthoughts or specifications (except for direct objects),
- constituents which can only appear in the post-field as they have an antecedent in the middle-field which they semantically specify or elaborate, or
- syntactically non-integrated constituents that function as metacommentaries.

## 4. Results

### 4.1. Descriptives

Descriptive statistics show the mean percentages of LWC types in the post-field across speaker groups (Table 6), the absolute frequencies of LWC types in the post-field across speaker groups and narratives (Table 7) and the mean percentages of LWCs in the post-field across speaker groups and narratives (Table 8).

**Table 6:** Mean percentages of LWC types in the post-field across speaker groups.

Constituent type	Mean percent in HSs	Mean percent in MSs
PP	13.81	9.84
DP	2.86	1.81
DM	0.92	5.02
ADVP	2.86	2.01
ADJP	2.38	0.40
NONC	0.95	0.00

**Table 7:** Absolute frequencies of LWCs in the post-field across speaker groups and narratives.

Narrative	Spokenformal		Spokeninformal		Writtenformal		Writteninformal	
Speaker group	HS	MS	HS	MS	HS	MS	HS	MS
PP	16	25	5	11	8	4	0	5
DP	3	3	1	4	1	0	1	2
DM	0	2	2	21	0	0	0	2
ADVP	2	3	1	5	2	1	1	0
ADJ	0	1	2	0	3	1	0	0
NONC	1	0	1	0	0	0	0	0

**Table 8:** Mean percentages of LWCs in the post-field across speaker groups and narratives.

Narrative	Speaker group	Mean percent of LWCs
Spoken formal	HS	30.5
Spoken formal	MS	21.8
Spoken informal	HS	24.5
Spoken informal	MS	31.8
Written formal	HS	19.4
Written formal	MS	5.4
Written informal	HS	11.8
Written informal	MS	13.8

### 4.2. LWCs across speaker groups and narratives

**Figure 2:**
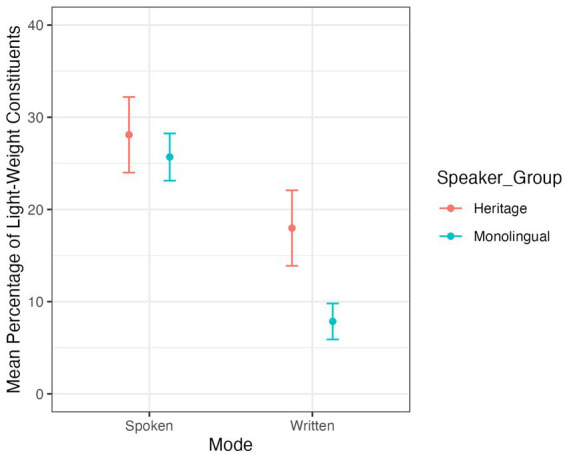
Mean percentage of post-field LWCs across speaker groups and modes.

**Figure 3:**
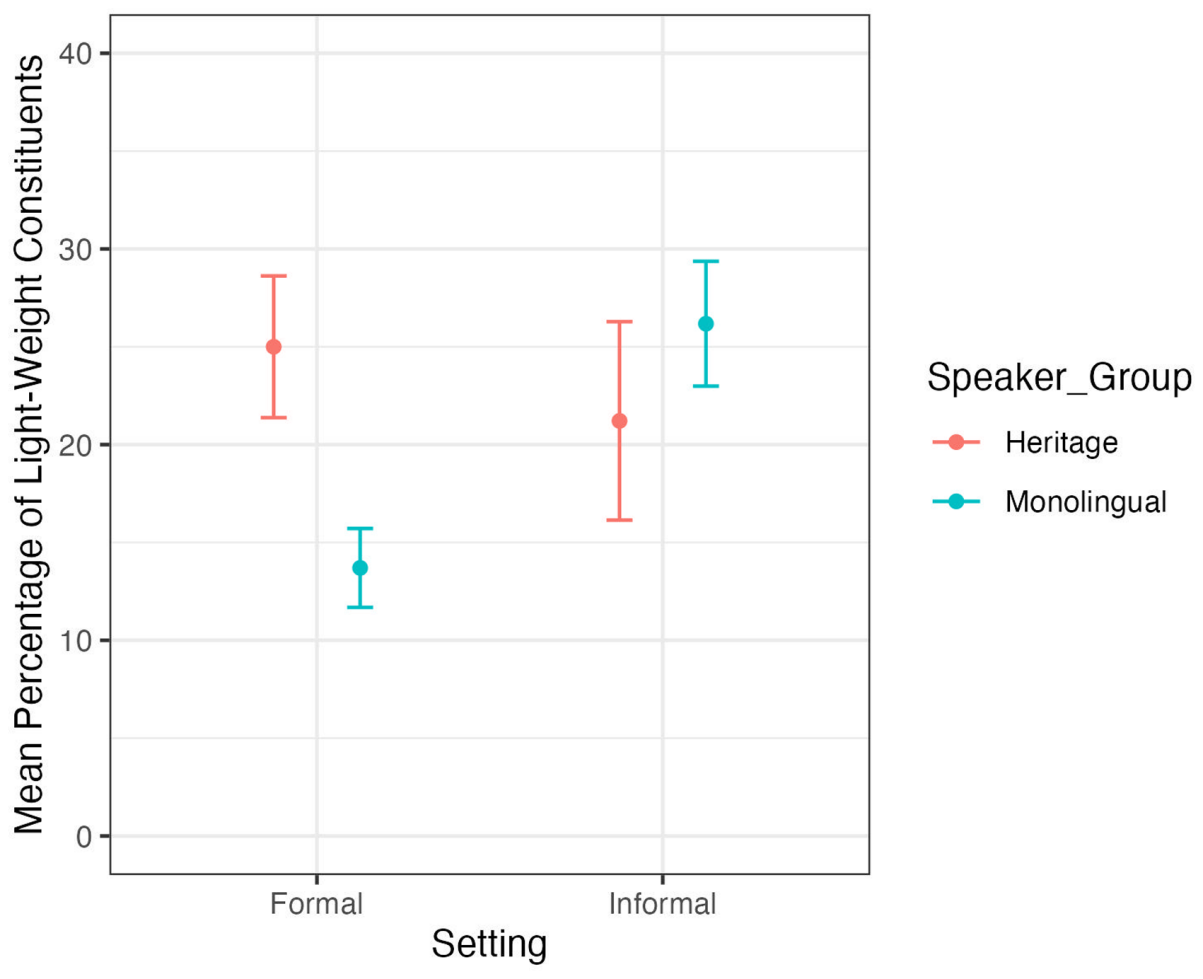
Mean percentage of post-field LWCs across speaker groups and settings.

For the frequency of post-field LWCs, the model output (Appendix A) shows no significant difference between the two speaker groups (*z* = −1.173, *p* = 0.241). For the distribution of LWCs in the post-field across registers (i.e., settings and modes), the model output (Appendix B) shows a main effect of mode (*z* = −4.677, *p* < 0.01, Figure 2) with both speaker groups producing more post-field LWCs in spoken productions than in written productions, independently of the setting. The model additionally shows an interaction between speaker group and setting (*z* = 3.226, *p* = 0.001, Figure 3). To interpret this interaction, I ran Tuckey’s multiple comparison test using the *emmeans* package (Lenth, 2020). Tuckey’s multiple comparison test (Appendix C) revealed a significant difference between speaker group in the formal setting (estimate = 0.976, SE = 0.345, *z* = 2.831, *p* = 0.024) but no such difference in the informal setting (estimate = −0.559, SE = 0.429, *z* = −1.305, *p* = 0.56). This indicates that HSs and MSs overlap in their frequency and distribution of post-field LWCs in the informal setting but not in the formal setting. Furthermore, Tuckey’s multiple comparison test (Appendix C) also revealed a significant difference in the setting of the MSs (estimate = −0.769, SE = 0.257, *z* = −2.99, *p* = 0.0148, Figure 3). MSs produced significantly more post-field LWCs in the informal setting than in the formal setting. In the HSs data, there is no significant difference in the production of post-field LWCs across settings. This shows that while mode plays a role in the production of post-field LWCs across speaker groups, setting only has an influence on the productions of MSs.

### 4.3. PPs across speaker groups and narratives

**Figure 4:**
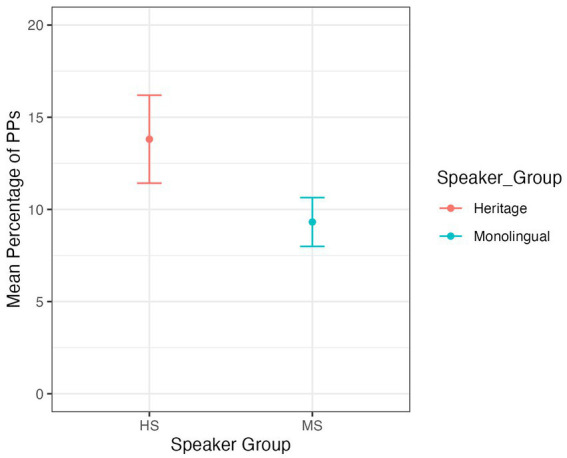
Mean percentages of PPs in the post-field across speaker groups.

For PPs in the post-field, the model output (Appendix D) shows no significant difference for the frequency of PPs between speaker groups (*z* = −1.506, *p* = 0.132, Figure 4). Hence, HSs and MSs do not differ significantly in their production of post-field PPs.

### 4.4. Non-canonical placement of direct objects in the post-field

The corpus presents two instances of NONCs in the post-field which can be attributed to the influence of the ML, English on the HL, German. We find these two instances in both the formal spoken and the informal spoken productions of one HS (see example 10a/b^21^).

(10a) und die mann geht zu helfen^22^ [**die mädchen**_**NONC**_] (**−**) **die essen** (**−**) **äh aufzuholen**^23^ (RUEG corpus formal spoken)

“*the man goes to help the girl pick up the food*”

(10b) diese mann: geht zu helfen [**diese** (**−**) **de: de frau_NONC_] die essen** (**−**)**au/**(**−**) **aufzuheben** (RUEG corpus informal spoken)

“*this man goes to help this woman pick up the food*”

The examples consist of two DPs and two infinitive clauses (INFs) each. In both cases, not only the direct object *die Mädchen* or *diese Frau* (the girl, this woman) but also the two infinitival constructions *zu helfen* (to help) and *die Essen aufzuholen/aufzuheben* (to pick up the food) are placed after the finite verb *geht* (goes). The extraposition of the second INF is not problematic and can be considered unmarked in German. Colloquially, the example sentences in (10a/b) could have been canonically produced as in example (10c).

(10c) der Mann geht der Frau helfen, **das Essen aufzuheben.**

“*The man goes to help the woman pick up the food*.”

What is problematic, and ungrammatical in German, however, is the switched position of the infinitive *zu helfen* and the direct object *die Mädchen* or *diese Frau*. As a consequence, the direct object surfaces post-verbally, where it would be expected in English. The influence of English is not only visible in the linearization of the constituents but also in how the infinitive is realized. In this case, due to the collocation *helfen gehen* (help go, go to help), the infinitival particle *zu* (to) must be left out.^24^

It appears likely, then, that English provided the clausal matrix in these cases and that we are dealing with a calque. Support for this claim can be found in three corresponding English narrations of the very same speaker (see examples 11a–c).

(11a) the man went to go help the lady pick up his food (RUEG corpus formal spoken)

(11b) the: guy he went to go help th(e)la(d)y pick (−) pick up the food (RUEG corpus informal spoken)

(11c) When he try to help the lady pick up her food (RUEG corpus informal written)

One further case of a seemingly highly marked LWC in the post-field is found in the formal written production of another HS (see example 12).

(12) Nichts ist passiert **zu die zwei Autofahrer**. (RUEG corpus formal written)

“*Nothing happened to the two drivers*.”

In German, *passieren* (happen) can be used with a dative complement with or without a PP (*etwas passiert* (*mit*) *jemandem_DAT_*, something happens with to somebody/something happens to somebody). What makes the pattern in (12) look like a calque from English, at first sight, may just be due to the choice of *zu* instead of *mit* (with). Had the participant written *Nichts ist passiert mit den zwei Autofahrern*, one would simply consider it unusual in a written narrative.^25^

## 5. Discussion

This study investigated the production of post-field LWCs in spoken and written productions of HSs and MSs of German, taking into account different registers. The goal was to determine how the two speaker groups deal with the options available to them under the same communicative tasks.

The first research question focused on types of LWCs produced in the post-field across speaker groups, and on their relative frequencies. The analysis of the data shows that, apart from two instances of clearly non-canonically placed direct objects in the post-field produced by one HS, all listed constituent types were found with overall similar frequencies in the post-field productions of both speaker groups. Hence, hypothesis 1, which stated that the productions of HSs will show a greater variety and a higher frequency of LWCs in the post-field, is not confirmed. HSs and MSs do not differ with respect to the frequency and variety of LWCs in the right sentence periphery. So, even though we are looking at an interface phenomenon, HSs adhere to German canonicity requirements: the head position in the VP and the placement of finite verbs in main and subordinate clauses, phenomena acquired early and relatively stable even under intensive language contact.^26^

The second research question focused on the influence of register (i.e., different modes and settings) on the frequency of LWCs in the post-field. With respect to MSs, the data confirms hypothesis 2. Setting and mode had an influence on the production of post-field LWCs in the MS group. MSs produced significantly more post-field LWCs in the informal setting than in the formal setting and they produced significantly more post-field LWCs in the spoken mode than in the written mode. With respect to the HSs, the data just partly supports hypothesis 2. Only mode had an influence on the production of post-field LWCs in the HS group. HSs produced significantly more post-field LWCs in the spoken mode than in the written mode. However, the data shows no difference between post-field LWCs in the informal and the formal setting. Hence, while there is no group-specific difference in the overall frequency and variety of post-field LWCs, HSs and MSs show different distributions across registers, resulting in larger production differences between HSs and MSs in the written mode and in the formal setting. This result aligns with previous findings which observed register levelling across different phenomena in HSs (Polinsky, 2018, pp. 323–324; Tsehaye et al., 2021; Pashkova et al., 2022 among others) and can be traced back to HSs’ limited exposure to communicative situations in their HL compared to their ML.

In order to test the influence of language contact and transfer more specifically, the third research question focused on the realization of PPs in the post-field. The goal was to investigate whether HSs of German produce more PPs in the post-field than MSs of German. The data does not confirm hypothesis 3, indicating that extensive contact with English does not lead to an increase in PP extraposition in HSs. This finding is not in line with the assumption that the availability of surface structure parallelism leads to an increase in converging patterns. Again, a possible explanation for this result might be that core syntactic features are acquired early both in monolingual children and simultaneous bilinguals (Müller and Hulk, 2000; Genesee, 2001; Gawlitzek-Maiwald and Tracy, 2005; Tracy, 2011 among others) and hence may prove to be particularly robust in HSs as well, even under increased contact with the ML and reduced contact with the HL. Another line of argumentation could be that we are witnessing language internal changes within German, with PPs being increasingly prone to extraposition among MSs.

The role of language contact and transfer was also addressed by a qualitative analysis of the two instances of NONCs in the post-field produced by a single speaker. The claim as to the influence of an English clausal pattern as the underlying matrix for these constructions has been corroborated by the English productions of this very speaker since they exhibit an identical pattern. These two instances, however, also indicate that even though a speaker produces non-canonical syntactic structures, these structures are systematic: they occur in two out of four German narrations and both times only in the spoken mode.

Concluding, we can say that the narrations produced by HSs and MSs exhibit different degrees of variation at the right sentence periphery. These differences, however, do not seem to be primarily due to bilingualism, language contact, or transfer, as we only find very marginal evidence (two cases in total) for NONCs in the post-field and no difference in PP productions. This finding is even more remarkable as we also find occasional non-canonically placed direct objects in the post-field productions of monolingually-raised German children (Elsner, 2015). It is therefore the role of register variation or, rather, register-levelling that becomes apparent in the HSs data which leads to distributional differences between the two speaker groups.

Limitations of this study include the relatively small sample size of the different post-field constituents which did not allow for a more fine-grained quantitative analysis of the distribution of different types of LWCs. Moreover, the overall length of narrations per speaker and the constituents in the middle-field have not been taken into account. This could have influenced the results in two ways. Firstly, shorter, less detailed narratives provide less opportunity for the extraposition of constituents, plus the self-ratings of the HS group indicate lower proficiency in the written mode, which, in some cases, coincided with shorter written productions. Secondly, no conclusions about the overall number of constituents which have been placed in the post-field in proportion to those realized in the middle-field has been drawn. An additional limitation can be found in the research design. This study relied on the standardized elicitation of quasi-naturalistic productions and not on an experimental task geared to the elicitation of post-field items. Additionally, the elicitation task of recounting a car accident in as much detail as possible facilitated the production of LWCs in the post-field as participants tended to add further detail where they felt more information might be needed, like in the police report. Further research with different elicitation scenarios, including turn-taking, could enhance the production of a wider range of post-field LWCs and more diversified discourse functions.

## 6. Conclusion

This article investigated the linearization of constituents at the right sentence periphery in narrative productions of adolescent HSs of German and MSs of German. More specifically, the frequency of post-field LWCs in different registers was analyzed in order to shed further light on the variational spectrum found at the right clausal edge. Bilingualism, language contact, register variation, and internal dynamics were investigated as possible sources of variation. Analyses showed a similar variational spectrum of constituent types and their frequencies in HSs and MSs. Furthermore, HSs and MSs behaved similarly regarding the frequency and type of LWCs across modes, providing evidence that post-field LWCs are still more of a spoken phenomenon. The analyses for setting, however, showed effects of register-levelling in the HS group, as, unlike MSs, they did not differentiate between formal and informal settings. This suggests that diverging awareness of register norms due to different input conditions is the source of distributional differences observed rather than transfer from the dominant language.

Previous studies have considered PPs to be particularly affected by language contact and transfer. This, however, was not the case here, as the two speaker groups did not differ in their overall productions of PPs. But most importantly: While we find more variation in the right sentence periphery in different registers in the productions of HSs, the overall grammaticality of clausal syntax is not in jeopardy. Therefore, in the light of research on language change and language contact, we can say that the data discussed does not show evidence that heritage German is changing from an OV to a VO structure. Constituents placed right are still placed right.

## Data availability statement

The data presented in this article is openly accessible via the RUEG corpus: https://zenodo.org/record/5808870.

## Ethics statement

The studies involving human participants were reviewed and approved by the Deutsche Gesellschaft für Sprachwissenschaft ethics committee and the Institutional Review Board (IRB) of the University of Maryland at College-Park. Written informed consent to participate in this study was provided by the participants’ legal guardian/next of kin.

## Author contributions

The author confirms being the sole contributor of this work and has approved it for publication.

## Funding

The research results presented in this publication were funded by the German Research Foundation (DFG) as part of the research unit *Emerging grammars in language contact situations: a comparative approach* (FOR 2537) in project P5 (project no. 394995401, GZ TR 238/5-1). The publication of this article was funded by the University of Mannheim.

## Conflict of interest

The author declares that the research was conducted in the absence of any commercial or financial relationships that could be construed as a potential conflict of interest.

## Publisher’s note

All claims expressed in this article are solely those of the authors and do not necessarily represent those of their affiliated organizations, or those of the publisher, the editors and the reviewers. Any product that may be evaluated in this article, or claim that may be made by its manufacturer, is not guaranteed or endorsed by the publisher.

## Footnotes

^1^Current research calls for further distinctions and additional fields, such as the pre-forefield, the extended post-field, and the right outer field (Zifonun, 2015), which only play a marginal role in the later discussion of this article.

^2^This refers to one of the four narrations (formal spoken, formal written, informal spoken, informal written) which the participants were asked to produce. Section 3.2 provides a detailed explanation of the herein applied method for data collection.

^3^The right sentence bracket can be realized or realizable (see Vinckel-Roisin, 2012, p. 144).

^4^Especially long relative clauses, which are placed in the middle-field create a considerable distance between the subject and the finite verb, which makes them hard to process.

^5^With the exception of resultative or directional predicates (e.g., Er hat es geschnitten [in kleine Stücke] (He has cut it into small pieces), Haider, 2010, p. 191).

^6^For the participants presented in this research, no larger HL speaker community outside the family is present. Some participants, however, report regular visits to relatives in Germany.

^7^See Haider (2010) for hierarchical approaches in post-field analyses.

^8^Even though relative pronouns and relative adverbs also lead to VL clauses, they are not placed in the LSB. One line of argumentation is that relative pronouns and relative adverbs, unlike complementizers, function as constituents and are, thus, placed in the forefield (Wöllstein, 2014, p. 27ff.; Imo, 2016, p. 214).

^9^From a generativist perspective, researchers still discuss the source of constituents appearing in the post-field (extraposition vs. base-generation). Some argue that movement as the source of extraposition is lacking in its explicatory nature (Haider, 2010), while others even go as far as saying that there is no movement to the right in German (Frey, 2015).

^10^Spoken and written productions can be seen as part of a conceptual continuum. This means that, depending on the situation and the context, written productions can become conceptually spoken (e.g., a diary entry) and spoken productions can become conceptually written (e.g., a sermon, cf. Koch and Oesterreicher, 2012).

^11^Due to scarceness of datapoints in this corpus, no distinction between the narrow and extended post-field (or post-field and right outer field) is applied in the quantitative analysis.

^12^Discourse structuring devices, i.e., hesitations, pauses, and intonational breaks (or punctuation in written productions) can provide relevant cues to the degree of connectedness to the previous clause and can be used to distinguish between functional differences of constituents in the right sentence periphery (e.g., Altmann, 1981; Frey, 2015 and the references therein; Imo, 2015).

^13^The functional exploitation of the postverbal position is already visible in German-speaking children’s early multiword utterances (Tracy, 1991, p.187).

^14^Diachronic analyses of the post-field show that (direct) objects are found in the post-field without jeopardizing the grammaticality of the sentence up until the era of New High German (Hinterhölzl, 2004; Coniglio and Schlachter, 2015).

^15^PPs can also be topicalized in English, thus occurring before the subject (e.g., on the table, she placed a vase). In German, topicalization of PPs is also possible. The PP would then, however, be placed in the forefield (e.g., auf den Tisch platzierte sie eine Vase). This serialization would be ungrammatical in English (i.e., *on the table placed she a vase). Similar surface syntactic patterns in English are residual and restricted to transitive verbs (e.g., on the table stood a vase) and presentational there-constructions, both highly dependent on the preceding context.

^16^As we also know from code-switching research, parallel surface structures may ease language mixing (Poplack, 1980; Muysken, 2000).

^17^One adolescent HS did not enter their birthdate, therefore, the mean and standard deviation for the HS group was calculated for 28 participants only.

^18^Participation requirements were that the HSs were either born in the U.S., or moved there before age two. The HS participants should not have received bilingual education but may have participated in German “Saturday schools” or other German-speaking activities. Speakers of established German Language Islands were excluded from the study.

^19^Questionnaire for adolescent participants of *Research Unit Emerging Grammars*: https://osf.io/qhupg/.

^20^The variable SC now includes both, finite and non-finite subordinations in the quantitative analysis.

^21^The spoken and written productions in examples (10) and (11) were not corrected or normalized and the original orthography of the written productions was kept.

^22^The undisrupted productions of the first infinitive construction *zu helfen* can be interpreted as a sign that the speaker does not question the fact that the matrix verb “help” needs to be produced with the particle *zu*. The second infinitive constructions *aufzuholen/aufzuheben* is accompanied by an increased number of non-verbal elements. Determining whether this is due to word finding issues or the production of the infinitive goes beyond the scope of this paper.

^23^These are transcriptions of the spoken data that include non-verbal discourse elements, such as pauses “(−)”, ruptures “/”, prolongations “:”, and hesitations “äh”.

^24^The German infinitive, sui generis, depends on the matrix verb. It can be realized as an infinitive without the particle *zu*, an infinitive with the particle *zu*, or an infinitive with the particle *um zu*. In examples (10a/b), the matrix verb “help” does not require the addition of the particle *zu* in German. An alternative canonical option would be *der Mann geht, um der Frau zu helfen, das Essen aufzuheben*. I am aware that this is a radically reduced explanation of the German infinitive, but it is merely to show the three options of infinitive-formation in German.

^25^The non-canonical preposition in this example changes the semantics of the verb *passieren*, which may result in different interpretations (happen to somebody vs. happen with somebody). In the present analysis, this constituent was categorized as a PP.

^26^Stability and retention of verb placement, but with considerable interindividual variation, have also been attested in research on German Language Islands, such as Pennsylvania German (Westphal Fitch, 2011), Moundridge Schweitzer German (Hopp and Putnam, 2015) or Texas German (Boas, 2009).
