# Supplementary material for: Light-weights placed right: post-field constituents in heritage German
Source: Front Psychol. 2023 Aug 24;14:1122129. doi: 10.3389/fpsyg.2023.1122129 (PMC10499507; doi:10.3389/fpsyg.2023.1122129)
Supplement: Supplementary file 1 [file Data_Sheet_1.pdf]

**Appendix A: Frequency of post-field LWCs**

```
Random effects:
 Groups     Name        Variance Std.Dev.
 speaker_ID (Intercept) 0.4837   0.6955
Number of obs: 693, groups:  speaker_ID, 60
```

```
Fixed effects:
            Estimate Std. Error z value Pr(>|z|)
(Intercept) -1.2451     0.2253  -5.526 3.27e-08 ***
speaker_MS  -0.3376     0.2878  -1.173    0.241
```

Model Formula: model 1.9 = glmer(nonSC~speaker_group + (1|speaker_ID), family = "binomial", data=Data_Frame_Right_Periphery_HS_MS, control = glmerControl(calc.derivs=FALSE))
