# Supplementary material for: Light-weights placed right: post-field constituents in heritage German
Source: Front Psychol. 2023 Aug 24;14:1122129. doi: 10.3389/fpsyg.2023.1122129 (PMC10499507; doi:10.3389/fpsyg.2023.1122129)
Supplement: Supplementary file 2 [file Data_Sheet_2.pdf]

Appendix B: Distribution of LWCs in the post-field across registers

Random effects:

Groups	Name	Variance	Std.Dev.
speaker_ID	(Intercept)	0.531	0.7287

Number of obs: 693, groups: speaker_ID, 60

Fixed effects:

	Estimate	Std. Error	z value	Pr(>|z|)	
(Intercept)	-0.6069	0.2728	-2.224	0.02613	*
speaker_groupMS	-0.9764	0.3449	-2.831	0.00464	**
modewritten	-1.1276	0.2411	-4.677	2.92e-06	***
settinginformal	-0.7671	0.3949	-1.943	0.05206	.
speaker_groupMS:settinginformal	1.5358	0.4702	3.266	0.00109	**

Model Formula: model1.8 = glmer(nonSC~speaker_group + mode + setting + speaker_group:setting + (1|speaker_ID), family = "binomial", data=Data_Frame_Right_Periphery_HS_MS, control = glmerControl(calc.derivs=FALSE))
