# Supplementary material for: Light-weights placed right: post-field constituents in heritage German
Source: Front Psychol. 2023 Aug 24;14:1122129. doi: 10.3389/fpsyg.2023.1122129 (PMC10499507; doi:10.3389/fpsyg.2023.1122129)
Supplement: Supplementary file 3 [file Data_Sheet_3.pdf]

Appendix C: Tuckey's multiple comparison test

contrast	estimate	SE	df	z.ratio	p.value
HS,formal - MS,formal	0.976	0.345	Inf	2.831	0.0240
HS,formal - HS,informal	0.767	0.395	Inf	1.943	0.2103
HS,formal - MS,informal	0.208	0.346	Inf	0.601	0.9319
MS,formal - HS,informal	-0.209	0.429	Inf	-0.488	0.9618
MS,formal - MS,informal	-0.769	0.257	Inf	-2.990	0.0148
HS,informal - MS,informal	-0.559	0.429	Inf	-1.305	0.5600

Model Formula: pairs(lsmeans(model1.8, ~speaker_group*setting, adjust="tuckey"))
