# Supplementary material for: Light-weights placed right: post-field constituents in heritage German
Source: Front Psychol. 2023 Aug 24;14:1122129. doi: 10.3389/fpsyg.2023.1122129 (PMC10499507; doi:10.3389/fpsyg.2023.1122129)
Supplement: Supplementary file 4 [file Data_Sheet_4.pdf]

**Appendix D: Frequency of post-field PPs**

```
Random effects:
 Groups     Name        Variance Std.Dev.
 speaker_ID (Intercept) 0.2554   0.5054
Number of obs: 693, groups:  speaker_ID, 60
```

```
Fixed effects:
                 Estimate Std. Error z value Pr(>|z|)
(Intercept)       -1.8805     0.2335  -8.053 8.05e-16 ***
speaker_groupMS   -0.4516     0.2999  -1.506    0.132
```

Model Formula: model2.4 = glmer(PP~speaker_group + (1|speaker_ID), family = "binomial", data=Data_Frame_Right_Periphery_HS_MS, control = glmerControl(calc.derivs=FALSE))
